# Utilization of early detection examinations by children in Germany. Results of the cross-sectional KiGGS Wave 2 study

**DOI:** 10.17886/RKI-GBE-2018-100

**Published:** 2018-12-12

**Authors:** Claudia Schmidtke, Benjamin Kuntz, Anne Starker, Thomas Lampert

**Affiliations:** Robert Koch Institute, Berlin, Department of Epidemiology and Health Monitoring

**Keywords:** EARLY DETECTION EXAMINATIONS, CHILDREN AND ADOLESCENTS, HEALTH MONITORING, KIGGS

## Abstract

Examinations for early detection of diseases (called U-Untersuchungen in Germany) are among the most important prevention measures at childhood age. According to KiGGS Wave 2 data, participation rates are over 95% for most of these examinations. 99.7% and 99.6% of children, respectively, who had reached the recommended age for these examinations participated in the U1 and U2 examinations, 98.0% and 98.1%, respectively, the U8 and U9 examinations. Participation rates for children from families with low socioeconomic status and those with a two-sided migration background are slightly lower. A comparison with previous KiGGS waves shows that the utilization of early detection examinations has increased significantly over the last ten years. During this time, social differences which were previously pronounced have decreased.

## Introduction

Examinations for early detection of diseases (called U-Untersuchungen in Germany) are part of the most important prevention measures during childhood. The statutory health insurance catalogue includes the early screening programme which aims to detect development disorders and diseases early and, where necessary, to provide adequate measures for treatment [1]. In addition to rigorous examinations regarding the overall physical and mental health of a child, each early detection examination has an age-specific focus, such as hearing and visual capacities. Examinations are accompanied by primary prevention advice for example on vaccinations, diet and accident prevention [2].

Whereas the U1 and U2 examinations usually take place within the maternity clinic, the following examinations generally take place at a paediatric or general medical private practice. This article considers the examinations U1 to U9 that are recommended for children up to the age of six. Two further early detection examinations, U10 (7-8 years of age) and U11 (9-10 years of age) are scheduled to take place at primary school age, the J1 (12-14 years of age) and J2 (16-17 years of age) examinations are for adolescents. However, it should be noted that not all statutory health insurers cover the costs for the U10, U11 and J2 examinations.

All findings of the early detection examinations are documented in an examination record (called the yellow booklet) that maternity wards or midwives provide to parents immediately after birth. The booklet also contains a detachable card, which practices then use to document the participation in the U2 to U9 examinations by marking the date, and including the practice’s stamp and signature. Early detection examinations are scheduled for specific ages and should be attended within a relatively closed time frame (Table 1). Also for premature babies the stipulated examination periods have to be met. Premature birth is taken into account in the assessment of the results.

**KiGGS Wave 2**

Second follow-up to the German Health Interview and Examination Survey for Children and Adolescents

**Data owner:** Robert Koch Institute

**Aim:** Providing reliable information on health status, health-related behaviour, living conditions, protective and risk factors, and health care among children, adolescents and young adults living in Germany, with the possibility of trend and longitudinal analyses

**Study design**: Combined cross-sectional and cohort study

**Cross-sectional study in KiGGS Wave 2**

**Age range:** 0-17 years

**Population:** Children and adolescents with permanent residence in Germany

**Sampling:** Samples from official residency registries - randomly selected children and adolescents from the 167 cities and municipalities covered by the KiGGS baseline study

**Sample size:** 15,023 participants

**KiGGS cohort study in KiGGS Wave 2**

**Age range:** 10-31 years

**Sampling:** Re-invitation of everyone who took part in the KiGGS baseline study and who was willing to participate in a follow-up

**Sample size:** 10,853 participants

**KiGGS survey waves**

► KiGGS baseline study (2003-2006), examination and interview survey
► KiGGS Wave 1 (2009-2012), interview survey
► KiGGS Wave 2 (2014-2017), examination and interview survey

More information is available at

www.kiggs-studie.de/english

The article reports current figures for the participation in early detection examinations (U1 to U9) based on data from the second wave of the German Health Interview and Examination Survey for Children and Adolescents (KiGGS Wave 2, 2014-2017). With reference to the earlier surveys of the KiGGS study, we also discuss the development of participation rates during the last ten years.

## Indicator

The German Health Interview and Examination Survey for Children and Adolescents (KiGGS) is part of the health monitoring system at the Robert Koch Institute (RKI) and includes repeated cross-sectional surveys of children and adolescents aged 0 to 17 (KiGGS cross-sectional study) that are representative for Germany. The KiGGS baseline study (2003-2006) was conducted as an examination and interview survey, the first follow-up study (KiGGS Wave 1, 2009-2012) as a telephone-based interview survey and KiGGS Wave 2 (2014-2017) as an examination and interview survey. A detailed description of the methodology used in KiGGS Wave 2 can be found in New data for action. Data collection for KiGGS Wave 2 has been completed in issue S3/2017 as well as in KiGGS Wave 2 cross-sectional study – participant acquisition, response rates and representativeness in issue 1/2018 of the Journal of Health Monitoring [4, 5].

KiGGS Wave 2 measured the utilization of early detection examinations through a questionnaire which was filled out by parents, answering the question: ‘What early detection examinations did your child take part in?’ All responses on early detection examinations were recorded, including the U10 and U11 examinations, which, however, are not considered in the following. As children born outside Germany are often not able to take part in the first examinations, the analysis also only includes children born in Germany. Children who were younger than the admitted age tolerance at the time of surveying and therefore could theoretically still have participated in the examinations were also excluded from the analysis for methodological reasons [6]. Beyond the participation in individual examinations, the analysis also looks at the participation in the complete set of examinations from U3 to U9. U7a is not considered, because this examination was only introduced in 2008 and the results from KiGGS Wave 2 on utilization of all examinations were to be compared to the results of the KiGGS baseline study, which was conducted between 2003 and 2006. If additionally a further examination is missed, the examination series is considered as incomplete.

The analyses are based on data from 13,799 children and adolescents born in Germany (6,887 girls, 6,912 boys) aged 0 to 17, whereby the number of cases varies depending on the examination considered. Data on participation in the complete examination series refer to the age range from 7 to 13 years and stem from the data from 5,867 children and adolescents (2,893 girls, 2,974 boys). The results are presented as prevalences with 95% confidence intervals (95% CI) and stratified according to gender, socioeconomic status of the family and migration background.

In KiGGS Wave 2, the socioeconomic status (SES) was measured through an index based on the information the parents provided on educational background, occupational status and income situation (equivalised disposable income). Based on an index built using a point score that equally considers the three indicators, a distribution-based distinction is established according to which 20% of children and adolescents belong to the low (1st quintile), 60% to the medium (2^nd^-4^th^ quintile) and 20% to the high status group (5^th^ quintile) [7].

As a category, migration background is built on child/adolescent and parent country of birth and parent nationality. A ‘one- sided migration background’ means that one parent was not born in Germany and/or is not a German national. A ‘two-sided migration background’ is assumed when the child has migrated to Germany from another country and at least one parent was not born in Germany or does not have German nationality or when both parents were born outside of Germany or are not German nationals [8].

The calculations were carried out using a weighting factor that corrects deviations within the sample from the population structure with regard to regional structure (rural area/urban area), age (in years), gender, federal state (as at 31 December 2015), German citizenship (as at 31 December 2014) and the parents’ level of education (Microcensus 2013 [9]). P-values to demonstrate linear trends across the three KiGGS survey waves were calculated using univariate logistic regression and were based, moreover, on age-standardised prevalences (as at 31 December 2015). A statistically significant difference between groups is assumed when the corresponding p-value is smaller than 0.05, taking into account weighting and the survey design.

## Results and discussion

KiGGS Wave 2 data indicates that nearly all children in Germany participate in the early detection examinations. For U1 and U2, which take place immediately or a few days after birth, the participation rates are 99.7% and 99.6%, respectively (Table 2). Participation decreases only slightly over the course of the set of examinations and is still 98.0% for U8 and 98.1% for U9. At 92.6%, participation in the additional U7a which was introduced in 2008 is lower.

No statistically significant differences between girls and boys are found regarding participation in early detection examinations. With regard to SES, significant differences between low compared to medium and high status groups exist, however for most examinations these differences are in the order of one to two percentage points. Only for the U8 and U9 are the differences slightly over two percentage points. Like children from low status groups, children with a migration background are slightly less likely to participate in early detection examinations. However, the differences are only statistically significant for children with a two-sided migration background.

**Figure 1:**
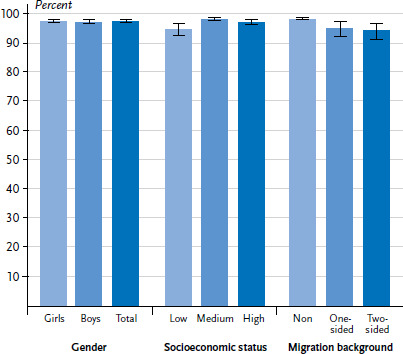
Participation in the complete set of early detection examinations U3 to U9 (without U7a) of 7-to 13-year-olds born in Germany according to gender, socioeconomic status and migration background (n=2,893 girls, n=2,974 boys) Source: KiGGS Wave 2 (2014-2017)

When analysing the participation in the total set of examinations (U3 to U9 without U7a), clearer statistically significant differences related to SES and migration background become visible (Figure 1). Of the 7- to 13-year-old children from families with low SES, 94.6 % participated in all examinations, while 98.0 % of their peers from families with medium and 97.0 % from families with high SES participated. 94.4% of children with a two-sided migration background participated in all examinations, compared to 95.1% of those with a one-sided migration background and 98.0% of children with no migration background.

Compared to the results of the KiGGS baseline study (2003-2006) and KiGGS Wave 1 (2009-2012), it is apparent that participation in early detection examinations has increased significantly over the past ten years [6, 10]. Participation in the full set of examinations has risen from 81.6% (2003-2006) to 82.2% (2009-2012) to currently 97.2%. At the same time, the differences according to SES and migration background, which were still strong in the KiGGS baseline study and KiGGS Wave 1, have decreased significantly.

The increasing participation in early detection examinations is confirmed by the results of the school entry examinations of public health services that require parents to bring the yellow booklet and/or the detachable card. For example, in the case of Brandenburg, the results indicate that the proportion of children who have made full use of examinations U1 to U8 has increased between 2004 and 2015 from 71.6% to 90.2% [11]. For North Rhine Westphalia participation in the U9 examination demonstrably rose from 82.6% to 93.4% between 2002 and 2012 [12]. In both federal states, this increase was accompanied by a decrease in social differences in participation rates.

The increased utilization of early detection examinations and also the reduction of social differences in participation rates can be linked to various measures. Since 2007, new invitation, reminder and feedback systems were introduced in all federal states, even though the regulations vary from one federal state to the other [13, 14]. In Brandenburg, for example, in cooperation with the ‘Bündnis Gesund Aufwachsen’, a registration system for the U6 to U8 was established in 2008 that commits doctors to confirm to a newly introduced central body, when the corresponding examinations have taken place. In North Rhine Westphalia a reporting system was created within the framework of ‘Aktion Gesunde Kindheit’ in 2008. Parents are also sent letters reminding them of upcoming examinations, although these measures were limited to U5 to U9. Further noteworthy measures that possibly caused participation rates in early detection examinations to rise include the awareness campaign ‘Ich geh’ zur U! Und du?’ conceived by the Federal Centre for Health Education (BZgA) and implemented between 2004 and 2010 [2]. By extending section 26 of Book 5 of the German Social Code (SGB V), statutory health insurers were moreover compelled to do more in order to promote utilization of early detection examinations. Since then, many statutory health insurers reward participation in early detection examinations as part of their bonus programs [6].

When interpreting the results, it should be borne in mind that the prevalences are based on self-reported information provided by the parents. In recent years, the importance of early detection examinations has been discussed and highlighted more in the public debate. In addition to the measures mentioned to promote child health, this process has been driven by the role of early detection examinations in the national health target process (gesundheitsziele.de) and in Germany’s Preventive Health Care Act (Präventions gesetz – PrävG). Against this backdrop, it cannot be ruled out that the marked increase in participation rates can be attributed to socially desirable response behaviour. As they assume that participating is what is seen as normal, some parents possibly will say their children have participated when they actually have not. This would explain why prevalences based on school entry examinations that require parents to bring the yellow booklet are slightly lower.

Finally, a frequent criticism of early detection examinations should also be mentioned. Participation in and quality of early detection examinations can only be scientifically analysed and evaluated based on a correct and complete documentation. A current analysis of examination results as recorded in the yellow booklets, which was conducted during the LIFE Child study at the University of Leipzig, for example indicates that information is often incomplete and frequently implausible. Inconsistencies exist particularly regarding psychosocial conditions [15]. The increase in early detection examination participation rates cannot conceal the fact that the collection and documentation of examination results as well as their use in epidemiology continues to present considerable challenges [15, 16].

## Key statements

- Participation rates for most early detection examinations are over 98%, and sometimes even over 99%.
- 97.2% of 7- to 13-year-old children have participated in all of the recommended examinations (U3-U9, excluding U7a).
- Participation rates for children from families with low socioeconomic status and those with a two-sided migration background are slightly lower.
- Utilization of early detection examinations has increased significantly over the last ten years.

## Figures and Tables

**Table 1:** Schedule and content of examinations U1 to U9 with margin of tolerance Source: Federal Joint Committee [3]

Examination	Time of examination (tolerance)	Content of examinations
U1	Immediately following birth	Detection of life-threatening complications and other conditions requiring immediate medical care, malformations, pregnancy, birth and family anamnesis, control of breathing, heartbeat, skin colour, signs of maturity
U2	3^rd^-10^th^ day of life (3^rd^-14^th^ day of life)	Detection of congenital anomalies and significant health risks, prevention of complications: anamnesis and examination of organs, sensory organs and reflexes
U3	4^th^-5^th^ week of life (3^rd^-8^th^ week of life)	Examination of normal development of reflexes, motor skills, weight and reactions, examination of organs, surveying of feeding, digestion and sleeping habits, examination of hip joints for hip dysplasia and luxation
U4	3rd 4^th^ month of life (2^nd^-4½^th^ month of life)	Examination of age-appropriate development and mobility, of organs, sense and sexual organs, the skin, control of growth, motor skills and the nervous system
U5	6^th^-7t^h^ month of life (5^th^-8^th^ month of life)	Examination of age-appropriate development and mobility, of organs, sense and sexual organs, the skin, examination of growth, motor skills and the nervous system
U6	10^th^-12^th^ month of life (9^th^-14^th^ month of life)	Examination of age-appropriate development and the organs, sense organs (in particular the eyes), control of the locomotor system, motor skills, language and interaction skills
U7	21^st^-24^th^ month of life (20^th^-27^th^ month of life)	Examination of age-appropriate development, detection of visual impairments, examination of language development, fine motor skills and body control
U7a	34^th^-36^th^ month of life (33^rd^-38^th^ month of life)	Focus on age-appropriate development of speech, early detection of visual impairments
U8	46^th^-48^th^ month of life (43^rd-^50^th^ month of life)	Intensive examination of development of language skills, pronunciation, behaviour, examination of mobility, coordination skills, reflexes, muscle power and tooth health
U9	60^th^-64^th^ month of life (58^th^-66^th^ month of life)	Examination of motor skills, hearing, vision and language development to identify and counteract any potential illnesses and disabilities before school entry

**Table 2:** Utilization of early detection examinations according to gender, socioeconomic status and migration background Source: KiGGS Wave 2 (2014-2017)

U1		U2	U3	U4	U5	U6	U7	U7a	U8	U9
(95 % Cl)		% (95 % Cl)	% (95 % Cl)	% (95 % Cl)	% (95 % Cl)	% (95 % Cl)	% (95 % Cl)	% (95 % Cl)	% (95 % Cl)	% (95 % Cl)
n=13.799		n=13.776	n=13.756	n=13.659	n=13.396	n=13.060	n=12.571	n=11.481	n=11.348	n=10.166
**Total (girls and boys)**	99.7	99.6	99.5	99.5	99.4	99.3	99.0	92.6	98.0	98.1
	(99.4-99.8)	(99.3-99.7)	(99.3-99.7)	(99.2-99.6)	(99.1-99.6)	(99.0-99.5)	(98.8-99.2)	(91.9-93.3)	(97.6-98.3)	(97.6-98.4)
Girls	99.7	99.6	99.5	99.5	99.4	99.2	98.9	92.6	97.7	98.0
	(99.3-99.8)	(99.2-99.8)	(99.1-99.7)	(99.1-99.7)	(99.1-99.6)	(98.7-99.4)	(98.5-99.2)	(91.7-93.5)	(97.1-98.2)	(97.3-98.5)
Boys	99.7	99.6	99.6	99.4	99.4	99.3	99.2	92.7	98.2	98.1
	(99.3-99.8)	(99.2-99.8)	(99.3-99.8)	(99.1-99.7)	(99.0-99.6)	(99.0-99.6)	(98.8-99.4)	(91.7-93.5)	(97.7-98.7)	(97.6-98.6)
**Socioeconomic status**										
Low	98.8	98.8	98.6	98.5	98.4	97.8	97.7	91.7	95.8	96.3
	(97.6-99.4)	(97.5-99.4)	(97.3-99.3)	(97.2-99.2)	(97.1-99.1)	(96.2-98.7)	(96.3-98.6)	(89.3-93.7)	(93.8-97.2)	(94.3-97.6)
Medium	99.9	99.8	99.8	99.7	99.7	99.6	99.4	92.9	98.5	98.5
	(99.7-99.9)	(99.6-99.9)	(99.7-99.9)	(99.4-99.9)	(99.5-99.8)	(99.4-99.8)	(99.2-99.5)	(92.1-93.7)	(98.1-98.9)	(98.1-98.8)
High	99.7	99.7	99.7	99.6	99.5	99.4	99.2	92.5	98.3	98.4
	(99.4-99.9)	(99.4-99.9)	(99.4-99.9)	(99.3-99.8)	(99.1-99.7)	(99.1-99.7)	(98.6-99.5)	(91.3-93.6)	(97.6-98.8)	(97.7-98.9)
**Migration background**										
Non	99.9	99.8	99.8	99.8	99.6	99.6	99.3	92.7	98.6	98.5
	(99.8-99.9)	(99.7-99.9)	(99.7-99.9)	(99.6-99.9)	(99.4-99.8)	(99.4-99.7)	(99.1-99.5)	(91.8-93.4)	(98.2-98.9)	(98.0-98.8)
One-sided	99.9	99.9	99.8	99.2	99.6	99.5	99.3	93.7	98.0	98.7
	(99.9-100.00)	(99.7-100.0)	(99.4-99.9)	(97.9-99.7)	(99.1-99.8)	(98.8-99.8)	(98.6-99.7)	(91.7-95.2)	(96.8-98.8)	(97.8-99.2)
Two-sided	98.1	97.7	97.5	98.0	97.8	97.0	96.9	91.2	94.3	95.2
	(96.4-99.0)	(95.8-98.8)	(95.6-98.6)	(96.2-99.0)	(95.9-98.8)	(94.9-98.2)	(95.0-98.1)	(88.5-93.2)	(91.6-96.2)	(92.3-97.0)

CI=Confidence interval
